# Genetics of Monogenic Diabetes: Present Clinical Challenges

**DOI:** 10.1007/s11892-018-1111-4

**Published:** 2018-10-30

**Authors:** Shivani Misra, Katharine R. Owen

**Affiliations:** 1 Diabetes, Endocrinology & Metabolism, Imperial College London, Ground Floor Medical School, St Mary’s Campus, Norfolk Place, London, W2 1PG UK; 2 Oxford Centre for Diabetes, Endocrinology & Metabolism, University of Oxford, Oxford, OX3 7LJ UK

**Keywords:** Monogenic diabetes, MODY, HNF1A, Genetic testing, Neonatal diabetes

## Abstract

**Purpose of Review:**

Monogenic forms of diabetes have specific treatments that differ from the standard care provided for type 1 and type 2 diabetes, making the appropriate diagnosis essential. In this review, we discuss current clinical challenges that remain, including improving case-finding strategies, particularly those that have transethnic applicability, and understanding the interpretation of genetic variants as pathogenic, with clinically meaningful impacts.

**Recent Findings:**

Biomarker approaches to the stratification for genetic testing now appear to be most effective in identifying cases of monogenic diabetes, and use of genetic risk scores may also prove useful. However, applicability in all ethnic groups is lacking. Challenges remain in the classification of genes as diabetes-causing and the interpretation of genetic variants at the clinical interface.

**Summary:**

Since the discovery that genetic defects can cause neonatal or young-onset diabetes, multiple causal genes have been identified and there have been many advances in strategies to detect genetic forms of diabetes and their treatments. Approaches learnt from monogenic diabetes are now being translated to polygenic diabetes.

## Introduction

Monogenic diabetes refers to a single gene defect that results in diabetes and is usually inherited in an autosomal dominant or recessive fashion. Monogenic diabetes can be broadly subclassified into neonatal diabetes, presenting before 6 months of age, and maturity onset diabetes of the young (MODY), presenting in youth and adults. Mutations causing diabetes may also be encountered in mitochondrial DNA and these are maternally inherited.

Estimates of MODY prevalence vary depending on the population studied and how they were stratified for testing. Whilst neonatal diabetes is rare, affecting approximately 1 in 100,000 births [1], MODY accounts for between ~ 1 and 4% of all cases of diabetes in those diagnosed under the age of 30 years [2•, 3].

The majority of MODY cases are accounted for by mutations in one of four genes: the transcription factors, hepatocyte nuclear factor 1-alpha (*HNF1A*), 4-alpha (*HNF4A*), 1-beta (*HNF1B*) and the enzyme, glucokinase (*GCK*) [4]. Since the discovery of these initial four genes as causes of MODY, rare mutations in other genes have also been identified, expanding the spectrum of genetic aetiologies in diabetes (see Table 1 [5]). In neonatal diabetes, the most commonly affected genes are *KCNJ11* and *ABCC8*, encoding the two subunits of the ATP-sensitive potassium channel of the beta cell (see Table 1) [1].

**Table 1:** Genes implicated in monogenic diabetes; MODY and neonatal diabetes

Gene	Protein	Function	Inheritance
Maturity onset diabetes of the young			
Common causes of MODY with well-established evidence-base			
*HNF1A*	Hepatocyte nuclear factor 1α	Beta-cell transcription factor	Autosomal dominant
*HNF4A*	Hepatocyte nuclear factor 4α	Beta-cell transcription factor	Autosomal dominant
*GCK*	Glucokinase	Glucose-sensor, first rate-limiting enzyme in glycolysis	Autosomal dominant
*HNF1B*	Hepatocyte nuclear factor 1β	Beta-cell transcription factor	Autosomal dominant
*ABCC8*	Sulphonylurea receptor subunit of β-cell K-ATP channel	Closure of the ATP-sensitive potassium channel leads to beta-cell membrane depolarisation, calcium influx and fusion of insulin secretory granules with beta-cell membrane	Autosomal dominant
*KCNJ11*	Potassium channel subunit of β-cell K-ATP channel		Autosomal dominant
*INS*	Insulin	Production of insulin or insulin action	Autosomal dominant
Rare causes of MODY with reasonable evidence supporting			
* NEUROD1*	Neurogenic differentiation factor 1	Beta-cell transcription factor	Autosomal dominant
*IPF1*	Insulin promotor factor 1	Beta-cell transcription factor	Recessive
*CEL*	Carboxyl ester lipase	Exocrine pancreas function	Deletion of variable number tandem repeat
*WSF1*	Wolframin	Function of the endoplasmic reticulum	Recessive
*RFX6*	Regulatory factor X 6	Beta-cell transcription factor	Dominant protein truncating variant
*APPL1*	Adaptor protein, phosphotyrosine interaction, PH domain, and leucine zipper containing 1	Protein that bind to AKT in the insulin-signalling pathway	Autosomal dominant
Neonatal diabetes			
Causes of neonatal diabetes, accounting for > 2.5% of cases			
*ABCC8*	Sulphonylurea receptor subunit of β-cell K-ATP channel	Closure of the ATP-sensitive potassium channel leads to beta-cell membrane depolarisation, calcium influx and fusion of insulin secretory granules with beta-cell membrane	Dominant, often de novo or recessive
*KCNJ11*	Potassium channel subunit of β-cell K-ATP channel		Dominant, often de novo
*GCK*	Glucokinase	Glucose-sensor, first rate-limiting enzyme in glycolysis	Autosomal recessive
*GATA6*	GATA binding factor 6	Transcription factor	Dominant, often de novo
*INS*	Insulin	Production of insulin or insulin action	Dominant, often de novo or recessive
*PTF1A*	Pancreatic associate transcription factor 1 A	Transcription factor involved in pancreatic development	Recessive
*EIF2AK3*	Eukaryotic translation initiation factor 2 alpha kinase 3	Kinase enzyme in endoplasmic reticulum	Recessive
*RFX6*	Regulatory factor X 6	Beta-cell transcription factor	Recessive

Some of the information in Table 1 came from https://www.diabetesgenes.org/tests-for-diabetes-subtypes/targeted-next-generation-sequencing-analysis-of-45-monogenic-diabetes-genes/” [5]

Making a diagnosis of monogenic diabetes has several benefits, which include: targeted treatment depending on the affected gene that is often superior to conventional approaches [6–8]; family member cascade testing to identify other, often misdiagnosed cases and prediction or early identification of other multisystem features that may be associated with the genetic defect.

Though the clinical benefits of a diagnosis are increasingly clear, significant challenges still remain. The first of these is to improve the identification of misdiagnosed cases as evidence suggests many are still misclassified [9]. In particular, a shift in focus towards the identification of MODY across all ethnic groups is warranted, as in certain ethnicities the overlapping features of young-onset type 2 diabetes and MODY are likely to obscure detection. Closely related to this is the need to improve accessibility of sequencing technologies and developing a standardised approach to select or stratify appropriate individuals for genetic testing, with consensus criteria that have transethnic applicability. Perhaps most importantly, though, is the need to ensure quality interpretation of genetic sequencing data that is clinically translatable and meaningful along with the need for consistency in classifying a gene as potentially causing monogenic diabetes.

In this review, we outline advances in the field of monogenic diabetes and discuss the remaining clinical challenges.

## Identifying Cases of Monogenic Diabetes

The biggest barrier to making a diagnosis of monogenic diabetes is lack of clinical suspicion or awareness. A UK study in 2012 demonstrated a marked geographical variation in referral to a centralised genetic testing laboratory [9]. Raising awareness of genetic forms of diabetes and recognising atypical features in people diagnosed with type 1 or type 2 diabetes, is therefore key. Though the cost of genetic testing continues to fall, it is still relatively expensive and testing is recommended in those individuals with a moderate to high possibility of a positive finding. Worldwide access to quality genetic testing services remains problematic.

### Clinical Approach

The original clinical criteria to define MODY included an age of onset below 25 years, not requiring insulin treatment and having a generational family history of diabetes [10]. Since this first description, however, the criteria have proven to be insensitive in many confirmed cases [2•], as well as increasingly non-specific. This is unsurprising with the rising prevalence of young-onset type 2 diabetes that is, at diagnosis, non-insulin requiring like MODY and typically occurs in the setting of a strong family history. Indeed, in some cases of adult-onset type 1 diabetes, the slow progression to beta-cell failure may not require insulin treatment immediately [11]; thus, the lack of a need for insulin is not particularly discriminatory for monogenic diabetes. Additionally, a family history of diabetes is often encountered in many people with any type of diabetes, again, due to the rising prevalence of type 2 diabetes. In addition it is recognised that > 20% of children with confirmed type 1 diabetes have an affected first-degree relative after decades of follow-up [12]. Age at onset below 25 years is also an arbitrary cut-off and, although commoner in younger adults, confirmed MODY mutations can present into the 5th decade of life [13].

In ethnic groups with a higher prevalence of young-onset type 2 diabetes, the discriminatory value of these criteria are even poorer. Indeed, in one study of UK south Asian individuals referred for genetic testing, although the south Asian referrals were more likely to meet the clinical referral criteria than white individuals, the detection rate of MODY in those genetically tested was less than half of the white group [14•].

The MODY probability calculator offers a more standardised approach to select individuals for genetic testing [15]. The online calculator combines broad clinical information to predict the probability of testing positive for MODY. When compared to traditional criteria, the prediction model improved the sensitivity and specificity for identifying MODY. However, the model was developed and validated in the same cohort of white individuals of European descent. There is a need for validation in other cohorts and some early studies in east Asians have shown good sensitivity but reduced specificity [16].

### Biomarker Approach

With non-specific clinical criteria, attention has turned to the use of biomarkers that assist the selection of individuals for genetic testing. C-peptide, pancreatic auto-antibodies, lipid profiles and high-sensitivity C-reactive protein (CRP) have variable discriminatory value, but also have several limitations [17–22]. Pancreatic autoantibodies are positive close to diagnosis in ~ 80% of people with type 1 diabetes if glutamic acid decarboxylase (GAD) and insulinoma antigen-2 (IA2) antibodies are measured [19], although titres may decrease with duration of diabetes, and a negative result does not therefore exclude type 1 diabetes. People from some ethnic groups with type 1 diabetes have been shown not to have detectable auto-antibodies at diagnosis, and so again the absence of pancreatic auto-antibody positivity does not preclude a diagnosis of type 1 diabetes.

C-peptide levels, a marker of endogenous insulin production, have also been used to help segregate people with type 1 diabetes (where C-peptide is low or undetectable) from those with MODY. However, this is not useful at diagnosis of diabetes, where C-peptide may be detectable in early type 1 diabetes, and even in long duration type 1 diabetes approximately 8% of people have stimulated C-peptide levels well above thresholds considered typical for type 1 diabetes [23]. Similarly, individuals with severe hyperglycaemia due to either monogenic or type 2 diabetes may have undetectable C-peptide levels due to transient beta-cell glucotoxicity.

Practically, a combination of clinical and biomarker approaches is likely to yield the highest sensitivity. In the US SEARCH study, youths diagnosed with diabetes below the age of 30 years were selected for genetic testing if pancreatic auto-antibody testing was negative and fasting C-peptide >0.8 ng/ml, and yielded a detection rate of 8% in those tested. In the Young Diabetes in Oxford study, a pick-up rate for MODY of 15% was observed in those with type 2 diabetes diagnosed before 45 years without features of insulin resistance or 10% of those with type 1 diabetes from individuals selected on the basis of antibody negativity and preserved C-peptide [24]. The UK UNITED study demonstrated a MODY prevalence of 3.6% using a biomarker approach in those diagnosed < 30 years with diabetes, a detectable urine C-peptide and negative antibodies. In this study, the MODY probability calculator also missed 55% of cases identified using the biomarker approach [25]. Recently, the Norwegian childhood diabetes registry was used to estimate MODY prevalence in all the antibody-negative children in the registry, which has a high case ascertainment. A total of 4.1% of individuals had likely pathogenic or pathogenic variants in the commonest MODY genes (*HNF1A*, *HNF4A*, *HNF1B*, *GCK* or *INS*) [3]. The biomarker approach is likely to result in more individuals being selected for testing. However, since the cases identified in those tested appear to be less likely to meet clinical criteria or are missed by the MODY probability calculator, testing more individuals may be warranted [2•].

### Genetic Risk Scores

When antibodies are negative and C-peptide levels are preserved, the clinical picture could be consistent with type 1, type 2 or monogenic diabetes. In these cases, assessing probability of type 1 or type 2 diabetes on the basis of polygenic risk, so-called genetic risk scores, may have some additional discriminatory value. A genetic risk score for type 1 diabetes that incorporates key human leukocyte antigen (HLA) susceptibility loci [26] has been shown to facilitate discrimination of type 1 from monogenic diabetes [27] and type 2 diabetes [28]. However, as with other approaches, this was validated in white European populations and evidence suggests ethnic-specific variations will be required [29].

In practice, misclassification of all diabetes types may occur and healthcare practitioners need be alert to this possibility [30, 31]. The application of a systematic approach to individuals who are newly diagnosed, particularly young adults and in those from non-white ethnic groups, could assist classification of common forms of diabetes and identify those in whom molecular investigation would be beneficial. Using biomarkers in this strategy is worthwhile as long as the limitations of these specialist tests are appreciated.

## Identifying Monogenic Diabetes Across Ethnic Groups

To date, monogenic diabetes has predominantly been studied in populations of European descent and systematic studies in other ethnic groups are lacking. In the UK, MODY was first reported in the south Asian ethnic group in a systematic survey of childhood diabetes [32]. A 2006 study revealed a lower than expected frequency of referrals for MODY testing in the south Asian ethnic group [33]. However, it was unclear if this reflected a genuinely lower MODY prevalence in this group or under-diagnosis due to referral bias and numbers were too small to explore the clinical characteristics of patients.

In a follow-up UK study from 2016, confirmed MODY mutations were observed in a variety of UK ethnic groups, including south Asian and African-Caribbean, though the detection rate was lower in these groups, compared to white [14•]. Interestingly, south Asian people referred for testing were more likely to meet clinical referral criteria.

The SEARCH study identified MODY mutations in African-American and Pacific Islander youths [34]. *HNF1A*, *HNF4A* and *GCK* mutations had been detected in various reported studies from India, although the assessment of what constitutes a pathogenic mutation versus a benign nucleotide change remains challenging [35–38]. Monogenic diabetes has also been detected in many east Asian populations, including China [39] and Japan [40].

Lessons can be learnt from a study of comprehensive testing for neonatal diabetes in 79 countries, which demonstrated variability in mutation frequency and inheritance patterns depending on the population studied [1]. For example, recessive *E1F2AK3* mutations were most common in countries with higher prevalence of consanguineous unions [1] whereas mutations in *KCNJ11* and *ABCC8* predominate elsewhere. A recent study in Oman demonstrates a higher frequency of recessive *GCK* mutations [41].

It is likely that the lower reported number of cases of MODY in non-white ethnic groups reflects a more challenging clinical separation from those with young-onset type 2 diabetes. For example, type 2 diabetes in south Asians is associated with a leaner body mass index (BMI) and stronger family history [42], and proportions of antibody positive individuals in non-white ethnicities with type 1 diabetes appear to be lower in native countries [43], although systematic assessment is lacking.

In preliminary data from the MY DIABETES study, the biomarker approach found similar detection rates of MODY across UK south Asian, African-Caribbean and white ethnic groups [44]. As the number of cases of young-onset type 2 diabetes continues to rise, and because obesity can co-present with any form of diabetes, including MODY, clinicians may have to expect to test more individuals to identify hidden cases of MODY, especially in ethnic groups with a high prevalence of younger-onset type 2 diabetes.

## Testing Individual Genes Versus a Panel

The facility to sequence known MODY-causing genes using targeted next-generation sequencing technologies has enabled the rapid recognition of mutations in people presenting with a MODY phenotype [45]. Molecular genetic testing has traditionally been guided by the clinical phenotype and also the relative prevalence of mutations within any given population [4]. In people with a very specific clinical phenotype, it might be pragmatic to request Sanger sequencing (a method that sequences a single gene in a single reaction) [46] of the selected gene alone; for example, *GCK* testing in a patient with isolated fasting hyperglycaemia, or *HNF1B* testing in a patient with renal cysts and diabetes [4].

In other types of monogenic diabetes, however, it may be difficult to predict the affected gene on the basis of clinical features alone. Previously, this situation would result in sequential testing of multiple genes, using Sanger sequencing. This could often result in long delays before a diagnosis was obtained. However, advances in DNA sequencing technologies have meant that now panels of genes can be tested simultaneously using next-generation sequencing platforms without the considerable costs and time associated with earlier sequencing approaches [1,45]. This approach mitigates somewhat against the circular paradigm of defining specific phenotype in individuals with associated genotypes, but only testing those individuals with the phenotype in the first place. Thus, it may be the case that individuals with the same mutation could present differently, but because only those with the ‘defined’ phenotype are being tested, those other individuals are not detected.

Studies using targeted next-generation sequencing (tNGS) approaches have found mutations in genes other than the common MODY genes. For example, in the UNITED study, 74% of known MODY cases had mutations in *HNF1A*, *HNF4A* or *GCK*. However, using a biomarker approach with tNGS, 47% had mutation in these genes [2•].

The availability of robust panels of diabetes genes that incorporate coding and potentially regulatory regions will be laboratory-dependent and is not widely agreed or available. However, limiting testing to the common MODY genes clearly misses cases of MODY in other genes that may have real impact on management, for example MODY mutations in *ABCC8* or mitochondrial diabetes, testing for which has been incorporated into some panels.

## Is the Variant Pathogenic?

Determining pathogenicity of a variant is now the biggest challenge in the interpretation of exome-based sequencing data and of huge importance in the clinical context, where incorrect interpretation can have serious clinical consequences [47]. Assigning pathogenicity is a particular issue in genes associated with monogenic diseases such as MODY, where distinguishing between variants that are clearly disease-causing versus those that impair protein function or are neutral, is problematic [48]. This issue is further complicated by the fact that many of the variants found in the common MODY genes are novel or arise de novo, making it impossible to gain insights from other affected individuals [48, 49].

A variety of guidelines, both national and international, have attempted to standardise the approach to investigating variant pathogenicity using in silico modelling, database searches and other parameters to assist with classification and to also standardise notation [47, 50, 51]. Variants are graded from 1 to 5; 1, benign; 2, likely benign; 3, variant of unknown significance; 4, likely pathogenic; and 5, pathogenic; and guidelines stipulate that variants classed between 3 and 5, should be reported to requestors (see Table 2).

**Table 2:** Strategies to establish pathogenicity of a variant in diabetes genes

Approach	Parameter	Description
In silico data	Genome database searches	Search of genome/exome databases e.g. GnomAD to establish mean allele frequency and also assess presence of other variants at affected nucleotide position
	Sequence variant databases	For example, dbSNP, Exome Variant Server or 1000 genomes NGRL
	Mutation database searches	Human Mutation Genetic Database search, imports published data on genetic mutations. Limitations, as mutations published may not necessarily prove to be pathogenic
	Amino acid change	Examines the effect of the amino acid substitution on charge and polarity. A significant change in polarity or charge from the amino acid substitution might be more likely to impair protein function
	Species conservation	Conservation can be scored using tools such as ConSurf, through which multiple sequence alignments can be undertaken to compare orthologs across species. Essential sites for protein function are likely to be invariant across species (highly conserved)
	Software prediction	Software prediction models: SIFT, PolyPhen2 and AlignGVGD Grantham Distance assessed the physico-chemical difference between amino acid properties
	Cryptic splice site	Online software that can predict whether the variant creates a cryptic splice site or alters an existing one
In vitro data	Cellular studies	For HNF1A, GCK and KCNJ11, published studies exist that demonstrate effects of variants on protein function. In turn, these can be fed back to affected individuals to establish best treatment options or management approaches. Lack of accessibility limits widespread use
Clinical data	Biomarkers/systemic features	Examining for features associated with the genetic mutation may help decipher whether a variant is disease-causing or benign. For example, high-sensitivity C-reactive protein levels are known to be lower in people with HNF1A MODY, so demonstrating undetectable levels in a person with a variant of unknown significance may be a helpful indicator to prove functional effects Low magnesium levels or evidence of pancreatic exocrine failure in people with HNF1B variants, is another example A history of neonatal hypoglycaemia and/or fetal macrosomia in HNF4A variants might be helpful In GCK variants, demonstrating only fasting hyperglycaemia or a history of fetal macrosomia may assist
	Treatment response	Demonstrating sensitivity to sulphonylurea therapy is a compelling piece of evidence favouring pathogenicity in HNF1A and HNF4A variants Other monogenic genes are not so easily readily identified in this way
	Co-segregation studies	Proving the variant segregates with diabetes in a family is compelling evidence in favour of pathogenicity. To achieve this robustly, in an affected kindred, people with diabetes should have the variant and those without the variant an absence of diabetes. Conclusive proof comes from co-segregation in a different kindred to the proband

### Database Searches

One such database is the Genome Aggregation Database (GnomAD), which contains data from over 100,000 exomes and 15,000 whole-genomes. GnomAD reports minor allele frequency (MAF) depending on ancestry. These databases can be used to assess whether a variant found in a dominant gene might be disease-causing. For example, the presence of a variant in more individuals than would be predicted from the population prevalence of MODY (50–100 per million [9]), assuming complete penetrance, would indicate that the variant is not acting as an autosomal dominant pathogenic mutation, since it would be present in people without disease.

However, knowledge of MAF is not a magic bullet. Each exome in GnomAD contains an average of 7.6 rare variants (MAF < 0.1%) in Mendelian disease genes, which suggests tolerance to genetic variation is higher than previously assumed. Such data have been used to downgrade pathogenicity status in variants that have previously been thought to be disease-causing [52].

The spectrum of low-frequency variation (MAF < 1%) in the seven commonest genes associated with MODY was examined in 4003 individuals from population studies [53]. Although 1.5% of those studied harboured genetic variants that had been previously reported to cause MODY, the majority were still euglycaemic through to middle age. This study was an early indication that careful consideration in assigning pathogenicity to variants in dominant genes, is needed.

### Species Conservation

Species conservation measures the degree to which amino acid properties at any given position are evolutionarily conserved [51]. Essential sites for protein function are likely to be invariant across species (highly conserved), whereas a variant sites across species can, by definition, accommodate a degree of substitution. Therefore, missense variants encountered at highly conserved sites are more likely to be pathogenic [54–57].

### In Silico Predictions

Using web-based applications, missense variants can be classified based on the predicted effects of the variant on protein function. The prediction obtained from a single method should not be taken in isolation. Broadly speaking, four main approaches exist: those based on sequence conservation methods; those assessing impact on protein sequences; protein structure; and physicochemical properties and splicing-predictions [51, 58].

Software programs such as SIFT (J. Craig Venter Institute, La Jolla CA) [59, 60], Align-GVGD (International Agency for Research on Cancer hosted by the World Health Organization [61, 62]) and PolyPhen-2 [63, 64] can combine a variety of assessments to predict pathogenicity.

Despite these aligned approaches, it is still often difficult to confidently assign pathogenicity. In these cases, further work may be necessary to shed light on variant functionality. These may be clinical studies in the individual or family members, or by use of reliable in vitro functional or biochemical assays that assess the effect of the variant in a cellular system model [48]. Even then, confident assignment of pathogenicity may not always be possible due to the limitations of these techniques.

### In Vitro Functional Studies

A series of assays have also been described to investigate transcription factor variants, such as those in *HNF1A,* based on published studies [65–67]. For the *HNF1A* gene, which has been studied extensively, these include an assessment of transactivation potential (the transcriptional activity of HNF1A), protein production, nuclear-cytosolic localisation and DNA-binding in cellular systems.

In vitro functional studies have also been successfully deployed in cases of neonatal diabetes to support precision-based treatment. In individuals with *KCNJ11* mutations causing neonatal diabetes, the success of sulphonylurea treatment is determined by the specific mutation, as not all affected channels are sensitive to blockade. Knowledge of specific mutant effects in vitro can therefore help clinicians decipher whether to try higher doses to achieve full blockade of the channel, or whether insulin is needed [68, 69].

Guidelines recommend collaboration with molecular laboratories in cases where functional effects of variants using established techniques prove inconclusive, but where there is real clinical impact in assigning status. However, this may be challenging for clinicians to access readily as most MODY gene variants have not been studied in this way and accessible pathways to support these efforts routinely may need to be developed.

In practice, for people with monogenic diabetes, treatments can be trialled empirically with or without confirmed mutation status, but the frustration and anxiety in failing to receive a conclusive diagnosis in those with variants of unknown significance is high and the psychological burden remains unexplored.

### Clinical studies

In many cases, the clues towards pathogenicity come from studying the proband and their family members in more detail. For novel variants, familial co-segregation studies that demonstrate the variant in relatives with diabetes, but absence in those without diabetes, is compelling evidence in favour of pathogenicity. Similarly, demonstrating sulphonylurea sensitivity in those with uncertain variants in *HNF1A* or *HNF4A* can also be very helpful.

### Is the Gene or Variant Really Causing Diabetes?

Aside from the common MODY genes described, a number of other genes have been implicated as causing diabetes. These include the beta-cell transcription factors *IPF1* and *NEUROD1,* and *PAX4*, *KLF11* and *BLK.* The genetic evidence for these genes causing penetrant autosomal dominant monogenic diabetes is weak and initial findings have not been borne out from large-scale sequencing panels of genes.

Extremely rare mutations in other genes have also been identified. For example, deletions shortening variable number tandem repeats in the *CEL* gene appear to cause diabetes and pancreatic exocrine dysfunction [70] and specific mutations in *POLD1* can result in severe insulin resistance syndromes [71]. Loss of function variants in *APPL1* have also been identified in two families from an exome sequencing study. Co-segregation studies are promising, but it will remain to be seen whether *APPL1* variants will be reported in other MODY [72].

Understanding the phenotypic spectrum of variants from benign to disease-causing in dominant genes is also key. Common variants in *KCNJ11* and *GCK* can lead to small changes in glucose homeostasis, whilst less common variants leading to a more severe functional impact cause MODY and neonatal diabetes. Another example is the low-frequency E508K variant in *HNF1A*, which in the Latino population appears to increase risk of type 2 diabetes [73] rather than causing monogenic diabetes. A similar finding has been observed in the Wolfram syndrome gene *WFS1,* which causes a MODY-like phenotype in a small number of families [74], but common variation in the gene is also associated with risk of type 2 diabetes.

As new data are gathered, revisiting existing genes can also be helpful. Protein truncating variants in the beta-cell transcription factor *RFX6* have now been reported to cause MODY with low penetrance [75]. Previously, however, only homozygous mutations in *RFX6* had been implicated in neonatal diabetes with gut and gallbladder anomalies and the role as a MODY-gene was questionable due to variable penetrance [76]. Protein truncating variants robustly co-segregate with diabetes, but with an increased age of onset compared to HNF1A-MODY, as only 27% had developed diabetes by age 25, compared to 55% of HNF1A-MODY cases [75].

### Management of Monogenic Diabetes

The management of monogenic diabetes is based predominantly on observational data. The main application of personalised medicine is in the use of sulphonylurea (SU) agents in *HNF1A/HNF4A*–MODY and in neonatal diabetes caused by mutations in K-ATP channel components. This is supported by a small randomised controlled trial of gliclazide versus metformin in *HNF1A*-MODY [77] and in observational data collected in cases of neonatal diabetes. Therefore, people with mutations in *HNF1A* or *HNF4A* causing MODY can be managed with low-dose sulphonylurea therapy, and those misdiagnosed may therefore potentially stop insulin injections or have tablet regimens rationalised. These precision-based treatments not only achieve good glycaemic control, but there is compelling evidence demonstrating that they are superior to conventional approaches [8]. There are case reports of decades of successful treatment on SU therapy in *HNF1A*-MODY, although clinicians also, not infrequently, see cases who do not respond well despite possessing an *HNF1A* variant that is clearly diabetes-causing (personal observation). The reason for this variation in treatment response remains unclear. However, a recent study has suggested that higher HbA1c, higher BMI and longer duration of diabetes at the time of transfer to SU therapy from insulin, may predict failure of SU monotherapy, making the case for early genetic diagnosis [78].

A study of liraglutide use in *HNF1A*- and *GCK*-MODY examined both clinical response and mechanistic aspects [79]. This study showed no significant difference in glucose lowering effect between liraglutide and SU treatment, but more hypoglycaemia with SU use, suggesting that the SU dose used was too high. The additional expense and inconvenience of injection therapy does not appear to justify routine GLP1 therapy in *HNF1A*-MODY, and it is unknown how effective these agents would be in the context of long duration MODY with secondary SU failure. Most clinicians use metformin or a dipeptidyl peptidase-4 inhibitor as second-line treatment although there is no particular evidence base. The effect of sodium-glucose transporter-2 inhibitor agents in HNF1A-MODY, who already have decreased expression of *SGLT2* and low renal threshold, was shown in a single dose study to induce greater glycosuria than in type 2 diabetes [80] and it is unknown whether this would lead to greater adverse effects or increased efficacy of the agents.

Permanent neonatal diabetes (PND) caused by mutations in *KCNJ11* or *ABCC8* can be managed with high-dose sulphonylurea therapy in > 90% of cases, negating the need for insulin therapy and associated complications in affected neonates [68]. For neonatal diabetes, a recent case series of 10-year follow-up of individuals with *KCNJ11* or *ABCC8* variants treated with SU agents reported that 93% of cases remained on SU therapy alone, achieving glycaemic targets and with a good safety profile [81].

*GCK* MODY, characterised by lifelong, non-progressive fasting hyperglycaemia, requires no pharmacological therapy and, following diagnosis, affected individuals may be able to stop all treatment. In the largest longitudinal study to date of people with *GCK* mutations, there is no higher burden of clinically significant microvascular or macrovascular complications, compared to normoglycemic individuals [82].

Most other forms of MODY, neonatal diabetes and mitochondrial diabetes are characterised by insulin deficiency and therefore, in most cases, individuals progress to insulin therapy. Those with mitochondrial diabetes tend to have increased lactate levels, which has raised concerns over metformin use, although there is no evidence that there is an increased prevalence of lactic acidosis, since metformin only appears to cause lactic acidosis in the setting of severe renal dysfunction [83]. The pragmatic consensus is that other oral agents should be used in preference to metformin, particularly in those with neurological features.

### Further Research

The drive to deliver precision-based approaches in diabetes undoubtedly underpin many of the recent advances in monogenic diabetes, although there is still much to achieve. A critical step, as discussed, is to develop and validate strategies for finding cases of monogenic diabetes that have transethnic applicability.

Supporting clinical practitioners to understand and deliver often complex genetic information to people with diabetes, is also a key area that lacks firm guidance. As accessibility to sequencing technologies expands, particularly in understudied populations, there is a potential harm from poor interpretation of variant pathogenicity that needs to be addressed. Close collaboration between clinical practitioners and centralised laboratories that undertake these activities may be helpful in this context.

Moving beyond monogenic diabetes and understanding the impact of common variants on type 2 diabetes risk, phenotype and treatment effects, is likely to be the next clinically meaningful direction for the lessons learnt from monogenic diabetes. Efforts to address this unmet need are underway and are very likely to change the approaches to management of common diabetes types [84].

## Conclusion

There have been many advances in the field of monogenic diabetes, although considerable challenges remain. Improving awareness of monogenic diabetes and accessibility of cost-effective and high quality genetic testing services remains a key challenge. Whilst stratified biomarker approaches to case-finding appear to be more successful than conventional clinical criteria, widespread applicability is limited due to the paucity of transethnic data. Much remains to be done to facilitate diagnostic pathways that are clinically safe, at a national level. A crucial step underpinning such efforts will be the validation of existing strategies across multiple ethnic groups, and potentially development of new strategies where current practices prove to be ineffective. The rising incidence of young-onset type 2 diabetes continues to obscure the detection of monogenic diabetes, although meaningful approaches to segregate these cases are unlikely to be forthcoming and practitioners may have to expect to test more individuals if all monogenic cases are to be found.
